# Influence of the Biotope on the Tick Infestation of Cattle and on the Tick-Borne Pathogen Repertoire of Cattle Ticks in Ethiopia

**DOI:** 10.1371/journal.pone.0106452

**Published:** 2014-09-23

**Authors:** Sándor Hornok, Getachew Abichu, Marina L. Meli, Balázs Tánczos, Kinga M. Sulyok, Miklós Gyuranecz, Enikő Gönczi, Róbert Farkas, Regina Hofmann-Lehmann

**Affiliations:** 1 Department of Parasitology and Zoology, Faculty of Veterinary Science, Szent István University, Budapest, Hungary; 2 National Research Center, Department of Parasitology, Arachnoentomology Unit, Sebeta, Ethiopia; 3 Clinical Laboratory and Center for Clinical Studies, Vetsuisse Faculty, University of Zurich, Zurich, Switzerland; 4 Institute for Veterinary Medical Research, Centre for Agricultural Research, Hungarian Academy of Sciences, Budapest, Hungary; University of Kentucky College of Medicine, United States of America

## Abstract

**Background:**

The majority of vector-borne infections occur in the tropics, including Africa, but molecular eco-epidemiological studies are seldom reported from these regions. In particular, most previously published data on ticks in Ethiopia focus on species distribution, and only a few molecular studies on the occurrence of tick-borne pathogens or on ecological factors influencing these. The present study was undertaken to evaluate, if ticks collected from cattle in different Ethiopian biotopes harbour (had access to) different pathogens.

**Methods:**

In South-Western Ethiopia 1032 hard ticks were removed from cattle grazing in three kinds of tick biotopes. DNA was individually extracted from one specimen of both sexes of each tick species per cattle. These samples were molecularly analysed for the presence of tick-borne pathogens.

**Results:**

*Amblyomma variegatum* was significantly more abundant on mid highland, than on moist highland. *Rhipicephalus decoloratus* was absent from savannah lowland, where virtually only *A. cohaerens* was found. In the ticks *Coxiella burnetii* had the highest prevalence on savannah lowland. PCR positivity to *Theileria* spp. did not appear to depend on the biotope, but some genotypes were unique to certain tick species. Significantly more *A. variegatum* specimens were rickettsia-positive, than those of other tick species. The presence of rickettsiae (*R. africae*) appeared to be associated with mid highland in case of *A. variegatum* and *A. cohaerens*. The low level of haemoplasma positivity seemed to be equally distributed among the tick species, but was restricted to one biotope type.

**Conclusions:**

The tick biotope, in which cattle are grazed, will influence not only the tick burden of these hosts, but also the spectrum of pathogens in their ticks. Thus, the presence of pathogens with alternative (non-tick-borne) transmission routes, with transstadial or with transovarial transmission by ticks appeared to be associated with the biotope type, with the tick species, or both, respectively.

## Introduction

Due to the veterinary-medical importance of hard ticks (Acari: Ixodidae) and costs of their control, the transmission of tick-borne diseases remains a challenge for cattle industry in the tropical and subtropical areas of the world, and it is a priority concern for many countries in these regions [1]. On the other hand, while modern molecular biological methods allow more effective detection of pathogens in tick species, these are expensive and require sophisticated laboratory instruments. As a consequence, although vector-borne (tick-borne) infections occur more frequently in the tropics [2], these are less frequently studied with up-to-date methods and relevant data are scarce in the literature.

Ethiopia has the largest livestock population in Africa [3], including 52 million cattle. Because ticks are widely distributed in the country [4], with more than 40 species of 10 genera [5], they severely affect cattle. Major tick-borne diseases in Ethiopia include anaplasmosis, babesiosi*s* and theileriosis [6]. In such a scenario it is utterly important to know those epidemiological factors, which may increase or decrease tick burdens of animals and thus the risks of tick-borne pathogen transmission. However, most regional studies report only the occurrence of tick species on Ethiopian cattle [7], [8], and molecular investigations are very few [9].

The farming system, the local cattle breed and the human population show marked differences throughout the various landscapes of Ethiopia. Human and livestock settlements have concentrated in the moist highland areas, whereas dry lowlands allow conditions for traditional nomadic life [3], [10]. In the face of such historical settings limited financial resources may explain why less attention was paid to compare epidemiological factors of tick-borne diseases between these highly divergent regions.

Thus the primary aim of the present study was to assess if (1) the tick infestation of cattle (i.e. tick species and/or their abundance), and (2) the presence of certain tick-borne pathogens/groups in ticks is influenced by the regional biotope used for grazing. In this context it was less important to consider here if PCR positivity implies a potential vector role for the relevant tick species in transmitting the detected pathogen. Instead, results focus on and are compared according to biotopes in which ticks could have acquired the evaluated pathogen(s), either from the current or one of their previous hosts.

## Materials and Methods

### Sample collection

The study area is situated in South-Western Ethiopia, along the Didessa valley (in the region between Nekemte and Jima, coordinates: 09°05′N, 36°33′E–7°40′N, 36°50E). Three types of habitats (biotopes) were selected for tick collection. These biotopes have different altitude, rainfall, relative humidity, temperature, vegetation coverage, wildlife, cultivated crops and livestock animals. Ticks were collected from cattle between June-July of 2012 in the following biotopes: (A) moist highland (above 1500 m altitude, in excess of 900 mm rain annually, temperature 18–20°C, with dense forest vegetation); (B) mid highland (less moist and cool, with mixed vegetation coverage showing altitudinal change); (C) savannah lowland (500–1500 m altitude, annual rainfall below 900 mm, temperature 18–24°C, with shrubs, gallery forests around rivers, woodland). Ticks were removed with strong pointed forceps from the skin of 109 cattle (35, 56 and 18 animals according to the above three biotope types, respectively) in 18 herds. Because this was part of the regular veterinary care and the field studies did not involve endangered or protected species, no specific permissions were required for these activities. All specimens were put into 70% ethanol in a separate vial according to host animal, and stored consequently at around room temperature.

### DNA extraction

*Amblyomma* specimens were prepared by mechanical removal of host tissues around their mouthparts, which are long and firmly cemented into the skin. All ticks were mechanically cleaned and their species identified according to [11], [12]. DNA was individually extracted from one specimen of both sexes (and if available, one nymph) of each tick species per cattle. Prior to DNA extraction these 295 specimens were taken out from the 70% ethanol, air dried, and individually washed sequentially in detergent-containing water, in tapwater and in distilled water. Air-dried ticks were then minced with pointed scissors at the bottom of Eppendorf-tubes, in 100 µl of phosphate-buffered saline (PBS). DNA was extracted using the QIAamp DNA blood mini kit (QIAGEN, Hilden, Germany) following the manufacturer's instructions and including an overnight digestion step (incubation at 56°C for at least 8 h) with tissue lysis buffer and Proteinase-K (QIAGEN, Hilden, Germany). Extractions included an extraction control to monitor cross-contamination of samples.

### Conventional PCR and sequencing for piroplasms

A 450 bp long portion of the *18S rRNA* gene of piroplasms was amplified with the primers PIRO-A1 [13] 5′-AGG GAG CCT GAG AGA CGG CTA CC-3′ and PIRO-B [14] 5′-TTA AAT ACG AAT GCC CCC AAC-3′. The reaction mixture contained 1×concentration of Coralload PCR Buffer, 1.5 mM of MgCl_2_, 0.2 mM of each dNTP, 25 pmol of each primer and 1 U of HotStarTaq Plus DNA Polymerase (QIAgen GmbH, Hilden, Germany) in a final volume of 25 µl. The reaction was run in a T-personal thermocycler (Biometra GmbH, Göttingen, Germany) according to the following program: initial denaturation for 5 min at 95°C was followed by a cycle 94°C for 30 s, 60°C for 30 s and 72°C for 40 s, repeated 35 times and finished with 72°C for 10 min. PCR products were visualized in 1.5% agarose gel prestained with ethidium-bromide. Strongly positive samples were selected for sequencing done by the Macrogen Inc. (Seoul, South Korea). Representative sequences were submitted to the GenBank (accession numbers KJ941104-12). In all PCR procedures positive (*Babesia canis* DNA) and negative controls (sterile deionized water) were included.

### Real-time PCR for *Coxiella burnetii*

The samples were screened using a sensitive and specific TaqMan real-time PCR assay for the IS*1111* element of *C. burnetii* [15]. The assay amplifies the superoxide dismutase gene and IS*1111* transposable element of *C. burnetii* with the primers 5′-CCG ATC ATT TGG GCG CT-3′ (forward) and 5′-CGGCGGTGTTTAGGC-3′ (reverse) and the probe 5′-6FAM-TTA ACA CGC CAA GAA ACG TAT CGC TGT G-MGB-3′, at concentrations of 1600, 800 and 200 nM, respectively. The reaction was started with 95°C for 10 min, followed by 40 cycles at 95°C for 15 s and 60°C for 60 s.

### PCRs and sequencing for rickettsiae

The presence of the members of spotted fever (SFG) and typhus groups (TG) rickettsiae was detected by using a previously published real-time TaqMan PCR assay specific for a 74-bp fragment of the citrate synthase (*gltA*) gene [16]. The PCR mixture contained a final concentration of 0.2 µM of primers (forward: 5′-TCG CAA ATG TTC ACG GTA CTT T-3′and reverse: 5′-TCG TGC ATT TCT TTC CAT TGT G-3′) and probe (5′-FAM-TGC AAT AGC AAG AAC CGT AGG CTG GAT G-BHQ-3′), 12.5 µl of 2×qPCR MasterMix Plus Low ROX (Eurogentec), and 5 µl or 2.5 µl of template in a final volume of 25 µl. The *gltA* assay was performed using 45 cycles on a ABI 7500 Fast Real-Time PCR system (Applied Biosystems), with an initial denaturation step of 20 s at 95°C, which was followed by 45 cycles of 95°C for 3 s and 60°C for 30 s [16]. In addition, from 21 samples with low threshold cycle (Ct) values the amplification of an approx. 750 bp fragment of the *gltA* gene and direct sequencing of the PCR product was attempted [17]. Representative sequences were submitted to the GenBank (accession numbers KJ941095-103). Sequences were edited and aligned with a consensus sequence using Geneious Version 7.1.5. For phylogenetic analysis, the sequences were aligned with known rickettsiae sequences from GenBank using ClustalW [18] and, if necessary, manually adjusted. Only the nucleotides available for all included sequences were used in the phylogenetic analysis. A bootstrap phylogenetic tree demonstrating the relationship between the isolates was created by the Neighbor-Joining method [19] using a distance matrix corrected for nucleotide substitutions based on the Maximum Composite Likelihood model. The dataset was resampled 1,000 times to generate bootstrap values. Phylogenetic and molecular evolutionary analyses were conducted using MEGA version 6 [20].

### Real-time PCR for haemotropic mycoplasmas (haemoplasmas)

The presence of haemotropic mycoplasmas or haemoplasmas was evaluated by using a universal screening assay based on the SYBR principle [21]. The primers were designed to fit the *16S rRNA* gene of haemotropic *Mycoplasma* species with known sequences. The reaction volume was 25µl, consisting of 12.5 µl of KAPA SYBR FAST qPCR Kit Master Mix (2x) Universal (KAPA Biosystems), a final concentration of 200 nM of forward primer (5′-AGC AAT RCC ATG TGA ACG ATG AA-3′), and an equimolar mixture (200 nM) of two reverse primers (5′-TGG CAC ATA GTT TGC TGT CAC TT-3′ and 5′-GCT GGC ACA TAG TTA GCT GTC ACT-3′), and 5µl of template DNA. Assays were performed using an ABI 7500 Fast Real-Time PCR system (Applied Biosystems). The SYBR green PCR protocol included an initial step at 95°C for 3 min, followed by 40 cycles of 95°C for 3 s and 60°C for 30 s. After the PCR run, dissociation was performed with the following thermal profile: 95°C for 15 s, 60°C for 1 min, a temperature increase from 60°C to 95°C with 1% gradient (for about 20 min), followed by 95°C for 15 s, and finally 60°C for 15 s.

### Statistical analysis

Abundance rates were calculated from the number of individuals of one species, expressed as the percentage of the number of all ticks. Sample prevalence data were analysed by using Fisher's exact test. Differences were regarded significant when P<0.05. Because of the large number of abundance/prevalence rates provided in the text below, exact confidence intervals are not shown.

## Results

### Abundance and habitat-dependent occurrence of ticks

Altogether 1032 ticks of seven species were collected: 1028 adults and four nymphs (Table 1). Concerning the adults, the most abundant was *Amblyomma variegatum* (558 out of 1028: 54.3%), followed by *A. cohaerens* (307 out of 1028: 29.9%), *Rhipicephalus decoloratus* (135 out of 1028: 13.1%), *Rh. evertsi* (17 out of 1028: 1,7%), *Rh. praetextatus* (8 out of 1028: 0.8%), *A. lepidum* (2 out of 1028: 0.2%) and *Hyalomma rufipes* (1 out of 1028: 0.1%). Among specimens of *Amblyomma* spp. the males predominated over females (752 vs. 115), whereas in case of *Rh. decoloratus* the contrary was true (male vs. female ratio was 9∶126).

**Table 1:** Distribution of tick species collected from cattle in three biotopes, and results of their molecular analyses.

biotope type	tick species^1^	all male/female specimens	number of ticks for PCR^2^	number of *Theileria* and *Babesia* positive samples^3^				number of PCR positive samples		
				*T. mutans*	*T. velifera*	*T. orientalis*	*B. caballi*	*Coxiella*	*Rickettsia*	*Haemoplasma*
**moist highland**	*A. variegatum*	100/11	35*	-	1^A^	1^A^	-	5	34	-
	*A. cohaerens*	68/14	**34**	-	-	2^A^	-	2	20	-
	*Rh. decoloratus*	5/35	**21**	3^A^ 2^B^	2^A^	4^A^ 1^C^	-	2	1	-
	other *Rh.* spp.	7/7	**14**	3^B^	-	1^A^	-	3	3	-
**mid highland**	***A. variegatum***	410/35	**81**	2^A^ 2^B^	2^A^	1^A^	2	4	80	1
	*A. cohaerens*	102/30	**51**	2^A^	2^A^	3^A^	-	2	33	1
	*A. lepidum*	2/0	**2**	-	-	-	-	-	2	-
	*Rh. decoloratus*	4/91	29*	8^A^ 1^B^	4^A^	1^A^ 1^B^ 1^C^	-	-	10	2
	other *Rh.* spp.	5/5	**10**	-	-	1^A^	-	-	2	1
**savannah lowland**	*A. variegatum*	0/2	2**	1^B^	-	-	-	2	-	-
	***A. cohaerens***	70/23	**15**	1^B^	1^B^ 4^C^	-	-	12	4	-
	other *Rh.* spp.	0/1	**1**	-	-	-	-	-	-	-
	***in toto***	773/254	**295**	15^A^ 10^B^	11^A^ 1^B^ 4^C^	14^A^ 1^B^ 2^C^	2	32	189	5

1 Tick species significantly more abundant in a biotope type, than in other(s), are marked with bold character. Abbreviations are “*A*.” for *Amblyomma*, “*Rh*.” for *Rhipicephalus*. Other *Rhipicephalus* spp. imply *Rh. praetextatus* and *Rh. evertsi*.

2 The number of asterisks (* or **) indicates the number of nymphs included in a sample number.

3 Upper index capital letters (A, B, C) on the numbers of *Theileria* positive samples indicate genotypes (for legend see Tables 2–4). *Theileria* genotypes unique to a tick species are marked with underlined superscript.

The *Hyalomma rufipes* male was PCR negative in all tests, therefore not shown.

According to biotopes, *A. variegatum* was significantly more abundant on mid highland (445 out of 686 ticks: 64.9%), than on moist highland (111 out of 248 ticks: 44.8%) (P<0.001). On the other hand, *A. cohaerens* was significantly more abundant on savannah lowland (93 out of 98 ticks: 94.9%), than on either moist highland (82 out of 248 ticks: 33.1%) or mid highland (132 out of 686 ticks: 19.2%) (P<0.001). No *Rh. decoloratus* was found on savannah lowland. Pathogens detected in ticks are summarized in Table 1.

### Piroplasms in ticks

*Theileria mutans*, *T. velifera* and *T. orientalis* were detected with prevalence rates of 8.5% (25 out of 295), 5.4% (16 out of 295) and 5.8% (17 out of 295), respectively. In two *A. variegatum* males *Babesia caballi* was also demonstrated. This strain had the highest, but only 97% sequence similarity to a *B. caballi* isolate from South Africa (EU642514).

The presence and prevalence of *Theileria* spp. in ticks (and most likely in the cattle from which they had been removed) was apparently not related to the biotopes, as *Rh. decoloratus* was significantly (P<0.001) more frequently *Theileria*-positive, than *A. variegatum* (or *A. cohaerens*) on both moist highland (12 out of 21 vs. 2 out of 35) and mid highland (16 out of 29 vs. 7 out of 81) (Table 1).

However, *Theileria* positivity seemed to be related to tick species, because the overall prevalence was significantly (P<0.001) higher in *Rh. decoloratus* (28 out of 50: 56%), than in *Amblyomma* specimens (25 out of 220: 11.4%). Concerning *Rhipicephalus* spp, *T. velifera* was identified only in *Rh. decoloratus*. Apart from this tick species, *T. orientalis* was detected only in *Rh. praetextatus*, and *T. mutans* in both *Rh. praetextatus* and *Rh. evertsi*.

There was a considerable sequence variation of all three above *Theileria* spp, with altogether eight genotypes, with differences in up to 16 nucleotide positions (Tables 2–4). The highest degree of sequence polymorphism was observed in the case of *T. mutans*, but *T. velifera* and *T. orientalis* had more genotypes in the samples. The numbers of ticks with the two genotypes (A and B) of *T. mutans* were more equilibrated, than for *T. velifera* and *T. orientalis* with the dominance of genotype-A in comparison with B and C (Table 1, bottom).

**Table 2:** Sequence differences of *18S rRNA* gene of *Theileria mutans* genotypes identified in this study, compared to GenBank reference sequences.

designation	nucleotid position in reference sequence (AF078815)															
	620	621	625	633	634	643	644	646	649	664	665	666	668	671	675	677
reference **(AF078815)**	A	T	G	T	C	A	G	G	T	A	C	T	G	T	T	T
genotype TM-A	•	•	•	•	•	•	•	–	•	•	•	•	•	•	•	•
genotype TM-B	C	C	C	–	–	G	A	–	C	C	G	A	–	C	C	G

**Table 3:** Sequence differences of *18S rRNA* gene of *Theileria velifera* genotypes identified in this study, compared to GenBank reference sequences.

designation	nucleotid position in reference sequence (AF097993)											
	620	627	628	629	637	644	646	648	650	652	653	677–8
reference **(AF097993)**	A	C	T	A	T	T	T	G	T	T	T	–
genotype TV-A	•	•	•	•	•	•	•	•	•	•	•	G
genotype TV-B	G	•	C	T	A	C	•	•	–	–	C	G
genotype TV-C	G	T	•	C	A	•	–	A	–	–	C	G

**Table 4:** Sequence differences of *18S rRNA* gene of *Theileria orientalis* genotypes identified in this study, compared to GenBank reference sequences.

designation	nucleotid position in reference sequence (AF236094)							
	626	630	637	639	654	676–7		
reference **(AF236094)**	A	A	G	T	T	–	–	–
genotype TO-A	•	•	•	•	•	•	•	•
genotype TO-B	•	•	•	•	•	T	T	A
genotype TO-C	T	T	–	C	C	–	–	–

In addition, it was noted, that if compared not on the species, but on the genotype level of the same *Theileria* spp, the B and C genotypes of *T. orientalis* were exclusively found in *Rh. decoloratus* (Table 1). Similarly, as B and C genotypes of *T. velifera* were unique to *A. cohaerens* (Table 1.), these could be detected only in association with savannah lowland. The genotypes of *T. mutans* were more evenly distributed among ticks species and biotopes.

### *Coxiella burnetii* in ticks

10.8% of ticks (32 out of 295) were positive for the Q fever agent (Table 1). PCR positivity was significantly (P<0.001) more frequently detected in ticks (collected from cattle) on savannah lowland (14 out of 18: 77.8%), than on mid highland (6 out of 173: 3.5%) or moist highland (12 out of 104: 11.5%). These data also imply, that *C. burnetii* was significantly more prevalent in ticks from moist highland, than in those from mid highland (P = 0.011). The biotope-related occurrence of *C. burnetii* is confirmed by the comparison of the same tick species in different biotopes, i.e., there was significantly higher PCR positivity among *A. cohaerens* ticks in savannah lowland (12 out of 15: 80%), than in either mid highland (2 out of 51: 3.9%) or moist highland (2 out of 34: 5.9%).

### Rickettsiae in ticks

**Figure 1:**
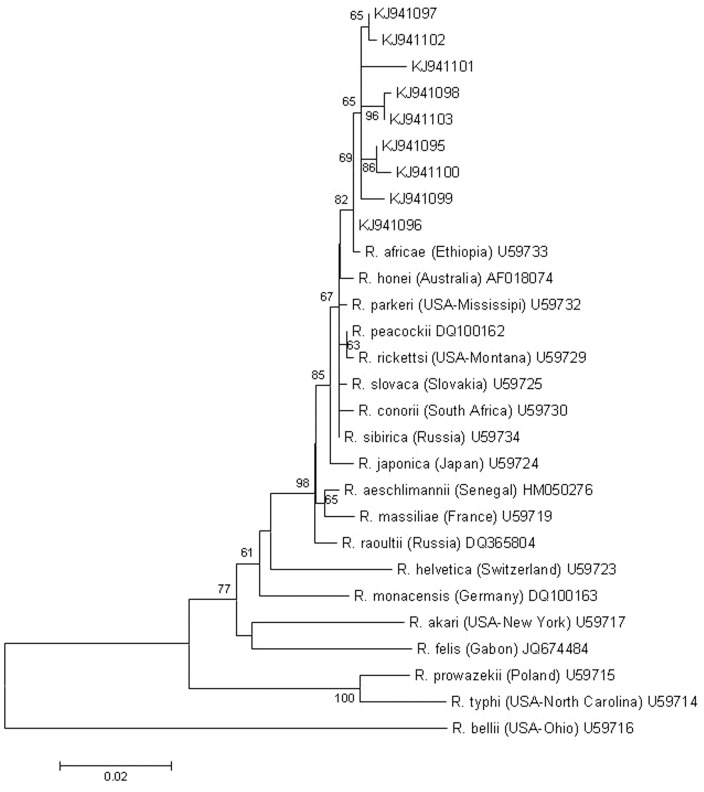
Phylogenetic comparison of *Rickettsia africae* isolates identified in the present study and other rickettsiae based on *gltA* gene sequences. Branch lengths correlate to the number of substitutions inferred according to the scale shown.

The overall prevalence of rickettsiae in ticks of the present study was 64.1% (189 out of 295). In the rate of rickettsia prevalence there was a significant (P<0.001) difference between the tick species: more *A. variegatum* (114 out of 118: 96.6%) and *A. lepidum* specimens (2 out of 2: 100%) were PCR positive, than those of *A. cohaerens* (57 out of 100: 57%); but PCR positivity was also significantly more frequently detected among individuals of the latter species, than among those of *Rh. decoloratus* (11 out of 50: 22%) or other *Rhipicephalus* spp. (5 out of 25: 20%) (Table 1). The presence of rickettsiae in ticks was also associated with biotopes in case of *A. cohaerens*: the prevalence was significantly lower on savannah lowland (4 out of 15: 26.7%), than on mid highland (33 out of 51: 64.7%) (P = 0.016) or on mid highland and moist highland taken together (53 out of 85: 62.4%) (P = 0.021). Similarly, *Rh. decoloratus* ticks significantly (P = 0.016) more frequently contained rickettsiae on mid highland (10 out of 29: 34.5%), than on moist highland (1 out of 21: 4.8%) (Table 1). In all 21 samples processed for sequencing *R. africae* was identified, with one to eight nucleotide differences to a reference sequence (U59733). The phylogenetic relationships of these isolates with each other and with other rickettsia sequences from the GenBank are shown on Figure 1. In summary, *R. africae* was detected in *A. variegatum*, *A. cohaerens* and *A. lepidum*.

### Haemoplasmas in ticks

The low level of haemoplasma positivity seemed to be equally distributed among the tick species (Table 1). The haemotropic *Mycoplasma* sp(p). in question could not be identified because of low bacterial loads (reflected by high Ct values). The presence of these pathogens was only detected in one type of tick biotope, i.e. on mid highland, but this association was not significant (P = 0.16) due to the small number of positive samples.

## Discussion

Hard ticks (Acari: Ixodidae) feed on the blood of vertebrates. Although they adversely affect domestic animals in several ways, their economically most important effect on domestic animals is connected to their vector role, as they are able to transmit a broad spectrum of tick-borne pathogens. The productivity losses attributable to related diseases are estimated to be highest in the tropical parts of the world, including Africa [22].

In order to infect new hosts, all tick-borne agents need the availability of certain ixodid species, i.e. competent vectors, which thus determine their geographical distribution. At a local scale, the epidemiology of tick-borne infections is strongly interrelated with the ecology of relevant ticks. In this way tick-borne diseases are usually endemic, implying their focal occurrence according to the suitable habitats of competent vectors.

Results on the abundance of tick species on cattle in the present study are similar to those reported earlier from South-Western Ethiopia, i.e. *A. variegatum*, *A. cohaerens*, *Rh. decoloratus* and *Rh. evertsi* being the most important ixodid species [7], [8]. The male-biased sex ratio of *Amblyomma* spp. and female-biased sex ratio of *Rh. decoloratus* is also consistent with previous findings [23]. However, here it was also demonstrated that the two most abundant tick species, *A. variegatum* and *A. cohaerens* are associated with mid-highland and savannah lowland, respectively, relevant to the endemicity and epidemiology of tick-borne pathogens they may carry.

*Theileria* spp. detected in ticks of cattle, i.e. *T. mutans, T. velifera* and *T. orientalis* are widespread in Africa and are usually regarded as mildly pathogenic. However, they were also shown to cause severe anaemia, icterus, even deaths [24]–[27]. Especially *T. mutans* have been associated with disease in cattle: invasion of brain capillaries by this piroplasm may result in a form of bovine theileriosis known as turning sickness [28].

In Africa these *Theileria* spp. are transmitted by ticks of the genus *Amblyomma* [28], [29]. This is consistent with the present findings, i.e. the relatively high prevalence of these piroplasms in ticks of cattle, particularly in *Amblyomma* spp. However, the fact that according to the results of this study *Rh. decoloratus* specimens also contained (had access to) all three above mentioned *Theileria* spp, it may deserve future attention to assess the vector competence of this tick species too. In addition, the prevalence of piroplasms (*Theileria* spp.) in cattle ticks was significantly higher (up to 56%) in the present study, than the 0.5–4% reported in Western Ethiopia recently [9], suggesting that their tick-borne transmission is more likely than previously thought.

A high degree of *18S rRNA* gene sequence polymorphism of these piroplasms was noted here, similarly to that reported in blood samples of the African buffalo (*Syncerus caffer*: the original hosts of *T. mutans*, *T. velifera* and *T. orientalis*) in South Africa [28]. Relevant to the epidemiological significance of the present findings, these genotypes are thought to circulate between some buffalo and cattle populations [28]. The present results also confirm that in comparison with *T. mutans* the sequence variation is less evident in the *18S rRNA* gene of *T. velifera* [28].

According to the data shown here the occurrence of (and risks associated with) these *Theileria* spp. and their genetic variants in South-Western Ethiopia are primarily dependent on the tick species (because they have specific competent vectors), and not on the type of tick-biotope where cattle are grazing. The latter may be explained by the absence of transovarial maintenance of *Theileria* spp. by ticks in nature [29], unlike in case of babesiae.

*Babesia caballi* is the large babesia of the horse, causing mainly anaemia. This piroplasm was detected in *A. variegatum* ticks, for the first in East Africa: a finding similar to the identification of the same piroplasm in cattle ticks in West Africa [30]. The sequence was new and highly (3%≤)divergent from already reported ones. Since cattle were an unlikely source of this piroplasm in the present study, the potential vector role of *A. variegatum* in transmitting *B. caballi* should be further evaluated.

*Coxiella burnetii* is a tick-borne zoonotic bacterium. Reports are scarce on its presence in ticks in East or West Africa. The Q fever agent is known to occur in Ethiopia for nearly half a century [31]. In a more recent study from West Africa [32] the infection rate with *C. burnetii* showed significant variations in ticks, e.g. in *A. variegatum* it was 0% in one region, and 37.6% in another, which phenomenon could not be completely explained by the authors, and particularly not with differences between tick biotopes. Ticks usually carry *C. burnetii* as reservoirs, and may transfer it between stages (transstadially) and even may inherit it to the next generation (transovarially). Nevertheless, ticks play a subordinate, most likely reservoir role in the epidemiology of Q fever [32], the most significant sources of infection remaining environmental: contact with animal faeces or products, or inhalation of similar substances following aerosolisation. As conditions (prerequisites) for the latter (i.e. dry weather, open area, wind) appear to be more available in savannah woodland, these alternative transmission routes may explain why ticks significantly more often carried (or had access to) *Coxiella* in this kind of biotope (taking into account that cattle were the most likely source of infection of ticks in the present study). Taken together, results presented here may be the first indications that the reservoir role of ticks is related to their habitats, i.e. could be more significant in savannah woodland, than in mid highland or moist highland.

In another West African study [33], also evaluating ticks from cattle, the prevalence of *C. burnetii* was higher (14% vs. 10.8%) and that of rickettsiae was significantly lower (12.5% vs. 64.1%) compared to findings in the present study. In an Ethiopian survey [34] the infection rate of ticks with rickettsiae was also considerably lower (4.1%). Therefore data obtained here, based on highly sensitive real-time PCR analysis, seem to attest for the first time, that rickettsiae may be present in the majority of *A. variegatum* and *A. cohaerens* ticks. In all samples of *A. variegatum* processed for sequencing *Rickettsia africae*, the causative agent of African tick-bite fever was found. This is in line with the known vectorial competence of *A. variegatum* in the transmission of *R. africae* [35]. However, *R. africae* was also identified in a few specimens of *A. cohaerens* and *A. lepidum*, justifying their future evaluation for vector role in the epidemiological cycle. Interestingly, the occurrence of rickettsiae in ticks of cattle, as shown here for the first time, may not only depend on the tick species (i.e. on their competent, specific vector), but also on the biotope type. This may be related to the transovarial maintenance of rickettsiae by ticks (bound to endemic foci) in nature [36].

Haemotropic mycoplasmas (haemoplasmas) are epi-erythrocytic bacteria that may cause anaemia and unthriftiness in various domestic animals or even humans, and were recently reclassified (removed from the order Rickettsiales). Despite *Eperythrozoon* (renamed as *Mycoplasma*) *wenyonii* was reported for the first time in Africa [37], more recent data are not available on the occurrence of haemotropic mycoplasmas in cattle or in ticks in Africa/Ethiopia. The present results not only attest for the first time the occurrence of cattle-related haemoplasmas in ticks collected in Africa, but also show that ticks preferring a more humid environment (in association with mid highland biotopes) may be more exposed to these agents. This biotope-related occurrence may also be partly explained by certain features of haemoplasmas on the group level, because the majority of species have non-tick-borne and other, alternative routes of transmissions [38], thus rendering them more dependent on environmental factors.

In conclusion, the tick biotope, in which cattle were grazed in the evaluated period, influenced not only the tick burden of these hosts, but also the spectrum of pathogens in their ticks. The biotope appeared to be an important limiting factor in case of tick-borne *Coxiella burnetii* and haemoplasmas, which are known to have alternative (non-tick-borne) transmission routes, but no specific tick vectors. The presence of rickettsiae in ticks was influenced by both the biotope type and the tick species, whereas that of *Theileria* spp. only by the latter. This may reflect, that representatives of these two pathogen groups have specific tick vectors, and in these rickettsiae also have the means (i.e. transovarial transmission) of long-term maintenance in nature.
